# Effect of difficulty of task on throwing performance and coping strategies in team handball

**DOI:** 10.3389/fspor.2023.1107861

**Published:** 2023-02-01

**Authors:** Roland van den Tillaar, Christopher Hope

**Affiliations:** Department of Sports Sciences, Nord University, Levanger, Norway

**Keywords:** accuracy, ball velocity, problem focused, emotional focused, avoidance

## Abstract

In this study the effect of level of opposition on throwing performance and coping strategies in the jump throw was examined in elite, amateur, and adolescent players in team handball. Twenty four participants consisting of 13 female elite junior handball players (age: 15.5 ± 0.7 years; height: 1.72 ± 0.07 m; body mass: 64.2 ± 7.0 kg; years of handball experience: 8.4 ± 1.76 years) and 11 senior recreational female handball players (age: 19.5 ± 1.04 years; height: 1.68 ± 0.08 m; body mass: 65.2 ± 9.3 kg; years of handball experience: 11 ± 2.61 years) performed ten jump throws under four conditions: (1) without opposition; (2) with a passive opponent; (3) with an opponent moving sideways; and (4) with a defender who was instructed to be unpredictable without physical contact with the thrower. Ball velocity and accuracy were measured for every throw together with answering a questionnaire consisting of 18 questions after each condition to investigate if coping strategies changed with increasing difficulty of task and if this was different for playing level. The main findings were that ball velocity and accuracy decreased when opposition was introduced, but with no differences when the opposition moved only sideways or unpredictably (forwards and/or sideways), similarly for both groups. Furthermore, the level had no influence on the coping strategies or a relationship with either of these coping strategies, but the avoidance coping strategy scored lower than the other two categories for both groups. It was concluded that level of opposition had a negative effect on throwing velocity and accuracy in elite junior and recreational level senior players which was probably caused by the change of given attention to one target (overcome opponent), which leaves less available for others (throwing velocity and accuracy). Furthermore, coping strategies did not change or have any correlation with throwing performance, indicating that these strategies seem to be influenced by trait and that most players mainly used problem- and emotional-focused coping strategies and less avoidance strategies when dealing with the level of opposition.

## Introduction

In team handball the main purpose is to score goals by throwing. Thereby, throwing velocity and accuracy are very important to surpass the goalkeeper. Various studies on experienced players in team handball have examined factors that influence throwing velocity and accuracy. Studies have investigated factors like throwing technique (1–6), anthropometrics (7–9), strength (10, 11), type of instruction (12–14), playing level (15, 16), playing position (17–20), fatigue (21, 22), gender (7, 23), and throwing direction (24). However, most studies are performed without any opposition (1–6), which could influence maximal throwing velocity. Only a few studies have investigated the effect of opposition on throwing velocity and accuracy (25–27).

While Gutierrez Davilla, Garcia (25) found no difference in throwing velocity when there was opposition, Rivilla-Garcia, Grande (26) found that an increase of opposition decreased throwing velocity and Zapardiel Cortés, Vila Suárez (27) found that without opposition, but with a goalkeeper the ball velocity was lower than with opposition. The discrepancy between the studies were caused by the set up. Both Gutierrez Davilla, Garcia (25) and Rivilla-Garcia, Grande (26) tested opposition in a training/lab situation, while Zapardiel Cortés, Vila Suárez (27) measured ball velocity during a world cup competition. In addition, Gutierrez Davilla, Garcia (25) and Rivilla-Garcia, Grande (26) only measured throwing velocity and not the accuracy of the throw, while Zapardiel Cortés, Vila Suárez (27) measured accuracy by the scoring effectiveness in goal or no goal. This effectiveness is dependent on the thrower, but also on the action of the goalkeeper. Thereby, the throws could be very accurate, but still miss effectiveness. Earlier studies have suggested that there could be a velocity accuracy trade-off. This velocity accuracy trade-off suggests that when focusing on accuracy velocity would decrease (28). Thus, with increasing opposition the accuracy of execution becomes more important (to overcome the block of the defense player, ball velocity would decrease) (26). However, this was not investigated before.

In addition, it is not known how players cope with these different levels of opposition. There are different coping strategies suggested that describe an individual's ability to manage stress in different ways and in different situations, with a bidirectional cooperation of one's belief of sufficient ability, both psychological and physiological (29). Coping strategies consist of three “higher order” possible strategies: (1) problem-focused coping, where the individual “attacks” the problem or the situation, eager to achieve a good result or eliminate the problem; (2) emotional-focused coping, where the individual focuses toward the feelings that arise in the given situation, and how to resolve the emotional distress, rather than the task at hand; and (3) avoidance coping, where internal dialogue reasons the individual to withdraw oneself from the situation due to too much stress (30). There is believed to be a vast difference between the three strategies, regarding result–outcome, especially in a sports' setting, with problem-focused coping being regarded as the best strategy for the best result, emotional coping strategy as the second best, and avoidance coping as the least convenient, although they are not mutually exclusive in their appliance, but one strategy dominates the situation (31).

Earlier studies on coping strategies in handball have shown that men have a higher level of confidence in their abilities and a higher level of motivation to deal with stressful situations than women (32) and that coping strategies are gender biased (33). Furthermore, the level of competitive experience seems to have a positive effect on stress coping (34). However, common for these studies is that they were not conducted in a controlled, measurable experiment, prior to completion of the questionnaire, alongside a standardized timeframe of completing the questionnaire afterwards. The potential problems of not having a measurable experiment to compare the questionnaire data is the risk of both the sporting event and the questionnaire becoming highly subjective in the evaluation. Regarding the different coping strategies and the relationship they have to performance, independent data collection with presentation of quantified numbers, as well as a limited amount of time between sporting context and implementation of the cognitive measuring instrument are required. In an area already assessing subjective data, perhaps it would be appropriate to use at least one measure of objectivity. Due to these shortcomings and the call for a more rigorous sampling of empirical evidence and the seeming dearth in the academic field of research, there is arguably a need for a new method for data sampling.

Therefore, the aim of the present study was to investigate the effect of level of opposition (difficulty of the throwing task) on jump throw performance (ball velocity and accuracy) and coping strategies in elite junior and recreational level female handball players. It was hypothesized that throwing performance decreases with increased difficulty of the task (26), while the coping strategies would change with increasing level of opposition in which the elite junior players would have a different coping strategy to the recreational level handball players (34). Furthermore, it was hypothesized that the more successful players (less decrease in ball velocity and accuracy) would have other coping strategies than the less successful players.

## Materials and methods

### Design

To answer the research questions, a repeated measures design was used in which throwing performance and coping strategies were tested in four conditions on level of opposition in a random order in an elite junior team and a recreational senior team followed by a questionnaire on coping strategies after each condition.

### Participants

Twenty-four participants consisting of 13 female elite junior handball players playing at the highest national division of their age category (age: 15.5 ± 0.7 years; height: 1.72 ± 0.07 m; body mass: 64.2 ± 7.0 kg; years of handball experience: 8.4 ± 1.76 years) and 11 senior recreational female handball players playing at the regional division (age: 19.5 ± 1.04 years; height: 1.68 ± 0.08 m; body mass: 65.2 ± 9.3 kg; years of handball experience: 11 ± 2.61 years) playing in outfield positions were recruited. Before participation, written consent was obtained from each of the participants. For participants under the age of 18 years, written consent was also obtained from the parents. The study was approved by the Norwegian Center for Research Data (NSD) and conformed to the latest revision of the Declaration of Helsinki.

### Procedure

To test the effect of level of opposition (difficulty of the task), a one-against-one test was constructed as a specific situation in the attacking play in handball, with the participants starting at 13 m and were instructed to attack the goal and finish with a jump shot from 9 m, with the goal of hitting a target with a diameter of 1 m, placed in the contralateral bottom corner of the preferred arm of the handball goal. The participants were instructed to hit the target as hard as possible, and to hit the middle of the target (12). The target consisted of five different scores with a 10 cm radius from the center of the target for each of the score areas (10 cm radius from the center of the target for the first, 20 cm for the second, 30 cm for the third, 40 cm for the fourth and 50 cm for the fifth). Before the test started, the participants were given five shots, without a defender to become familiarized with the test.

**Figure 1:**
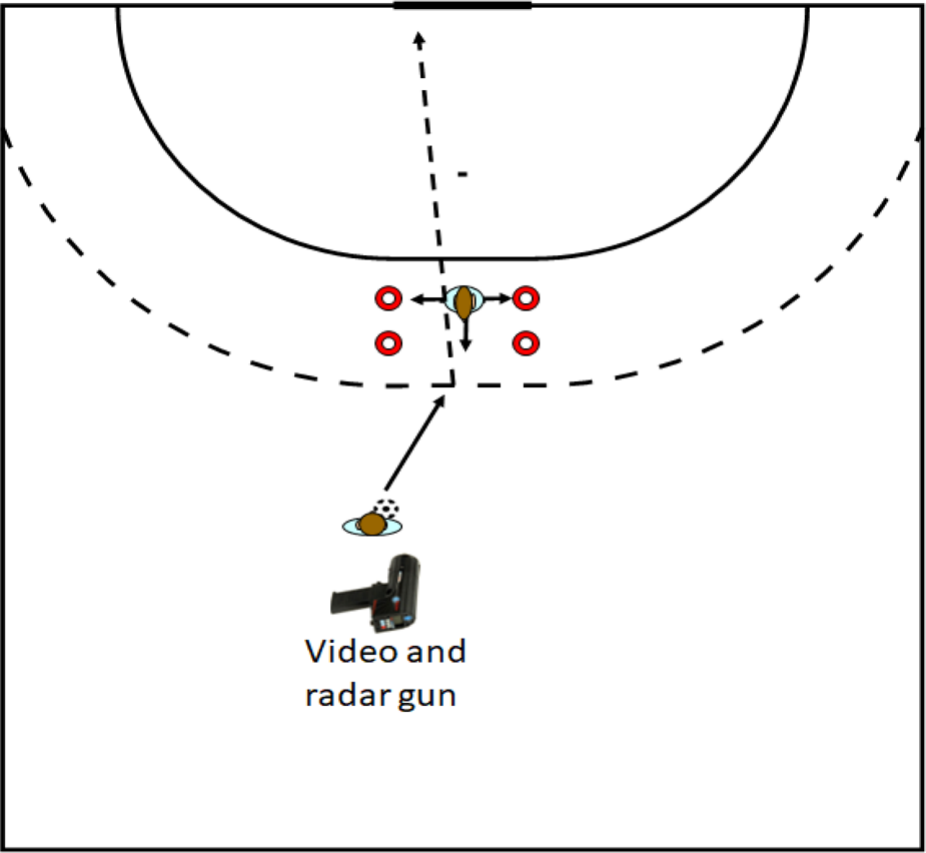
Experimental set up where participant performs a jump shot from 9 m distance with a defender (conditions 2–4) trying to block the shots with measuring devices.

The participants had to throw with a jump shot from 9 m distance under four different conditions of opposition varying in difficulty: (1) jump shot without defender in between; (2) with a passive defender standing on the 7 m line with arms over the head trying to block the shot; (3) with a defender moving sideways, following the attacker, on the 7 m line in a corridor of 2 m; and (4) with a defender who was instructed to be unpredictable in a field of 2 m × 1 m (side–forward) square starting from the 7 m line and not allowed to move out of the measured area (Figure 1). Since the defender was not allowed to go out of the area no physical contact between the participant and defender occurred. Each participant conducted ten attempts in each condition, and after every condition the participants had to answer the Coping Function Questionnaire (CFQ) according to the condition they were just exposed to. The conditions the participants completed where randomized using a random number generator to ensure the results were not compromised with ordering effects.

A Doppler radar gun (Stalker ATS II, Applied Concepts Inc., Plano, Texas) measured maximal ball velocity with ± 0.028 m/s accuracy, within a field of 10 degrees from the gun, at 15 m distance from the target located behind the participant at throwing height. Throwing accuracy was measured (50 Hz) with a video camera (Sony PXW-Z90, Sony, Tokyo, Japan) positioned 15 m from the target with full vison of the target and analyzed using Kinovea v. 0.9.3. (Kinovea.org). Accuracy was measured in two ways: (1) the score on the target (ranging from 1 to 5); and (2) distance from where the ball crossed the line/hit the target/goalposts to the center of the target. Attempts blocked by the defender were taken out of the calculation of the average velocity and accuracy per condition (Figure 1).

Coping strategies were measured using the CFQ questionnaire, which consists of 18 questions distributed over three higher order categories of coping: problem focused (six questions); emotional focused (seven questions); and avoidance coping (five questions) with a five-point Likert-scale. The comprehensive development of the CFQ by Kowalski and Crocker (35) confirmed the reliability and validity of the questionnaire. The average for each of the categories after each condition was used for further analysis.

### Statistical analysis

Means and standard deviations are presented for each group in every condition and coping strategy. A 2 (groups: junior, senior) × 4 (level of opposition: repeated measured) analysis of variance was performed on the throwing performance (accuracy and ball velocity) and coping strategies. When significant differences were found, a Holm–Bonferroni probability adjustment *post hoc* test was used to determine the source of those differences. Where the sphericity assumption was violated, the Greenhouse–Geisser adjustments of the *p*-values are reported. The effect size was evaluated with ${\eta_{p}}^{2}$ (Eta partial squared) where $0.01<{\eta_{p}}^{2}\;<0.06$ represents a small effect, $0.06<{\eta_{p}}^{2}<0.14$ a medium effect, and a large effect when ${\eta_{p}}^{2}>0.14$ (36). To investigate the relationship between level of opposition and coping strategies, the difference in throwing performance between the no opposition and highest level of opposition was calculated and a Spearman correlation with the three coping strategies was performed. The level of significance was set at *p* ≤ 0.05.

## Results

**Figure 2:**
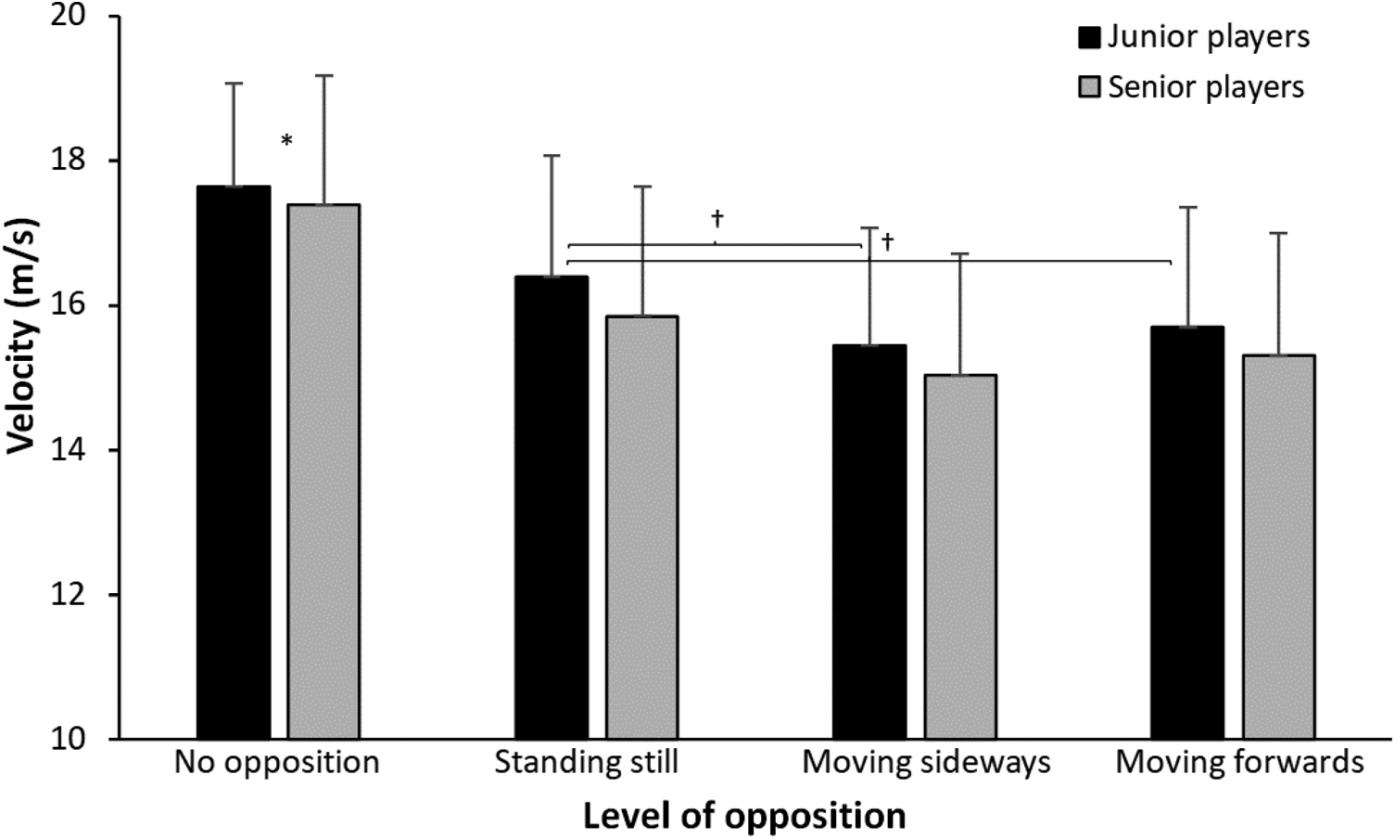
Mean (±SD) ball velocity over 10 attempts per group and level of opposition. * indicates a significant difference with all other levels of opposition for both groups on a *p* < 0.05 level. **†** indicates a significant difference in ball velocity between these two levels of opposition for this group on a *p* < 0.05 level.

A significant effect of level of opposition was found for ball velocity and throwing accuracy (F = 18.0, *p* < 0.01, ${\eta_{p}}^{2\;}>0.45$) with no significant difference between groups (F ≤ 0.46, *p* ≥ 0.50, ${\eta_{p}}^{2}\leq 0.02$) and interaction effects (F ≤ 0.43, *p* ≥ 0.73, ${\eta_{p}}^{2}\leq 0.02$). Post hoc comparisons revealed that the ball velocity decreased significantly when opposition was introduced. Furthermore, ball velocity decreased when level of opposition increased from standing still to moving sideways or forwards. However, this only reached significance level in the junior group (Figure 2).

**Figure 3:**
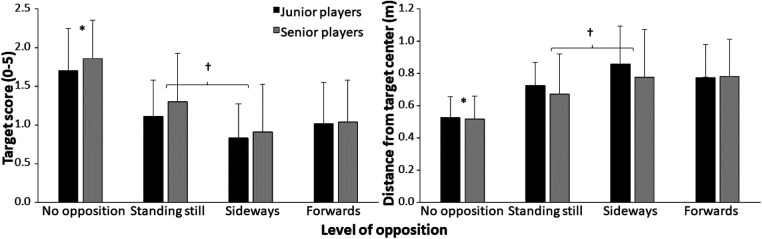
Average (±SD) target score and distance from target center over 10 attempts per group and level of opposition. * indicates a significant difference with all other levels of opposition for both groups on a *p* < 0.05 level. **†** indicates a significant difference between these two levels of opposition when both groups were taken together on a *p* < 0.05 level.

Accuracy also decreased when opposition was introduced. However, a further decrease in accuracy was only found between standing still and moving sideways when both groups were taken together (Figure 3).

No significant effects of level of opposition, between group and interaction on number of blocked shots were found (F ≤ 3.1, *p* ≥ 0.052, ${\eta_{p}}^{2}\leq 0.12$, Table 1). On average, one of the ten attempts was blocked in each level of opposition.

**Table 1:** Mean (±SD) number of blocked balls during the different levels of opposition per group.

Group	Standing still	Sideways	Forwards
Junior	0.54 ± 0.88	1.00 ± 0.82	0.69 ± 0.63
Senior	0.55 ± 1.04	1.36 ± 1.21	1.09 ± 1.22

**Figure 4:**
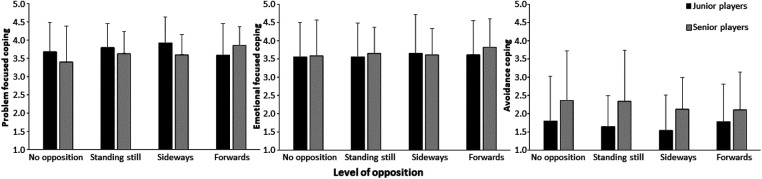
Average (±SD) **c**oping strategies for each condition for each group.

Furthermore, no significant effects of level of opposition (F ≤ 1.4, *p* ≥ 0.240, ${\eta_{p}}^{2}\leq 0.06$), group (F ≤ 1.6, *p* ≥ 0.214, ${\eta_{p}}^{2}\leq 0.07$), and interaction (F ≤ 1.6, *p* ≥ 0.187, ${\eta_{p}}^{2}\leq 0.07$) were found for any of the three coping strategies (Figure 4). A significant effect between the three coping strategy scores was found in which the participant scored avoidance strategy significantly (*p* < 0.01) lower than the other two categories (Figure 4).

Furthermore, no significant correlation between either accuracy (distance from target), ball velocity, and the three coping strategies was found (Table 2).

**Table 2:** Correlation between change in throwing accuracy (distance from center of target) and ball velocity with the three different coping strategies.

	Coping strategies		
	Problem focused	Emotional focused	Avoidance
Ball velocity	0.02	0.12	0.07
Accuracy	−0.15	0.03	−0.14

## Discussion

In this study, the effect of level of opposition on throwing performance and coping strategies in the jump throw was examined in elite, amateur, and adolescent players in team handball. The main findings were that ball velocity and accuracy decreased when opposition was introduced, but with no differences when the opposition moved only sideways or unpredictably (forwards and/or sideways), similarly for both groups. Furthermore, the level had no influence on the coping strategies or a relationship with any of the coping strategies, but the avoidance coping strategy scored lower than the other two categories for both groups.

No difference in any of the parameters was found between the elite junior and recreational senior players, which indicates that the level of the players was similar even when the playing level and playing experience were different. The decreased ball velocity with increasing level of opposition was in line with earlier studies in water polo (37, 38) and in handball (26) that included a goalkeeper and opponent as opposition. The decrease of ball velocity can be partly explained by the velocity accuracy trade-off proposed by Fitts (28), who stated that when the focus is on accuracy (in this case the accuracy to overcome the opponent), execution velocity and thereby, ball throwing velocity will be decreased. However, the accuracy also decreased when comparing the throws without opposition to those with opposition, indicating that more focus went into overcoming the opponents' action than the throwing accuracy at the target as proposed by Desimone and Duncan (39), who indicated that giving attention to one target (overcome opponent) leaves less available for others (shooting accuracy at target).

No difference in throwing performance was found when the opponent moved sideways or unpredictably, which was not expected as the level of difficulty was assumed to increase. An explanation for this absence of change may be the experience of the players. In training and competition, players are trained to perform a jump shot over the block of the opponent when the opponent moves sideways or forwards. In both situations, the opponent is moving, which increases the difficulty compared with the stationary opponent. The solutions to overcome the moving opponent seem to cost the same amount of attention and thereby, decrease the accuracy and throwing velocity by the same amount (39).

No effect of level of opposition and correlations with coping strategies were found, indicating that coping responses for sports, and specifically handball, could operate more on a trait-like level, rather than a state level, which agrees with the findings of Giacobbi jr and Weinberg (40) and Nicholls, Holt (41), who stated that coping most likely operates on a trait-level. Furthermore, no differences in coping strategies between the two groups were found indicating that some coping strategies are more important than others at different playing levels. It seems that both groups have to use the same coping strategies to solve the level of opposition and that problem-focused and emotional-focused coping strategies are the main strategies used in the jump throw task and that avoidance coping is a strategy that is not applied much by these participants.

The present study has some limitations as the participants were limited in the direction they could hit the target (contralateral corner down). This limitation restricted the maximal throwing velocity as throwing to the ipsilateral side results in higher ball velocities (24). However, this side was chosen since it is the most unexpected side for the goalkeeper. Furthermore, no goalkeeper was included, which also certainly would decrease the throwing velocity as previously shown by Rivilla-Garcia, Grande (26). However, including a goalkeeper would make it difficult to determine accuracy since a goalkeeper, by positioning in the goal, can influence the direction of throwing. In addition, there are several different coping strategies between and within participants that could result in the same performance outcome in an acute study. These coping strategies could change over time, i.e., a participant has missed many goals in the last games and could thereby be more directed to avoidance coping. However, this was not investigated in the present study, but it would be interesting to investigate if the coping strategies change over time within participants by using a longitudinal study to examine if the coping strategies are a trait or state ability in handball.

## Conclusion

It was concluded that level of opposition had a negative effect on throwing velocity and accuracy in elite junior and recreational level senior players which was probably caused by the change of given attention to one target (overcome opponent), which leaves less available for others (throwing velocity and accuracy). Furthermore, coping strategies did not change or have any correlation with throwing performance, indicating that these strategies seem to be influenced by trait and that most players used mainly problem- and emotional-focused coping strategies and less avoidance strategies when dealing with the level of opposition.

## Data Availability

The raw data supporting the conclusions of this article will be made available by the authors, without undue reservation.
